# Residential segregation, dividing walls and mental health: a population-based record linkage study

**DOI:** 10.1136/jech-2015-206888

**Published:** 2016-02-08

**Authors:** Aideen Maguire, Declan French, Dermot O'Reilly

**Affiliations:** 1 Centre for Public Health, Queen's University, Belfast, UK; 2 School of Management, Queen's University, Belfast, UK

**Keywords:** RECORD LINKAGE, MENTAL HEALTH, Neighborhood/place, EPIDEMIOLOGY, DEPRESSION

## Abstract

**Background:**

Neighbourhood segregation has been described as a fundamental determinant of physical health, but literature on its effect on mental health is less clear. While most previous research has relied on conceptualised measures of segregation, Northern Ireland is unique as it contains physical manifestations of segregation in the form of segregation barriers (or ‘peacelines’) which can be used to accurately identify residential segregation.

**Methods:**

We used population-wide health record data on over 1.3 million individuals, to analyse the effect of residential segregation, measured by both the formal Dissimilarity Index and by proximity to a segregation barrier, on the likelihood of poor mental health.

**Results:**

Using multilevel logistic regression models, we found residential segregation measured by the Dissimilarity Index poses no additional risk to the likelihood of poor mental health after adjustment for area-level deprivation. However, residence in an area segregated by a ‘peaceline’ increases the likelihood of antidepressant medication by 19% (OR=1.19, 95% CI 1.14 to 1.23) and anxiolytic medication by 39% (OR=1.39, 95% CI 1.32 to 1.48), even after adjustment for gender, age, conurbation, deprivation and crime.

**Conclusions:**

Living in an area segregated by a ‘peaceline’ is detrimental to mental health suggesting segregated areas characterised by a heightened sense of ‘other’ pose a greater risk to mental health. The difference in results based on segregation measure highlights the importance of choice of measure when studying segregation.

## Introduction

The physical and social characteristics of where we live can affect our health. One such characteristic, residential segregation, is acknowledged to be a fundamental determinant of physical ill health associated with risk of cancer, heart disease, obesity, low birth weight and increased infant mortality.^1–4^ The mechanisms underlying these associations are not well understood, but segregation is likely to play a significant role in determining access to resources such as education, employment, transport and healthcare which are all associated with health outcomes.^5^ Segregated areas are also associated with high rates of antisocial behaviour and crime prompted by intergroup contact, especially in the form of hate crime, sectarianism and crimes for financial gain.^6–8^

However, evidence for the effect of segregation on mental health has produced conflicting results. In a recent review of neighbourhood characteristics and risk of depression, only 4 out of 10 studies found an association between residential segregation and mental health.^9^ Furthermore, all of these studies were based in the USA or Canada, focused on racial residential segregation, with small samples of the population, and all involved self-reported measures of depression. None of the studies adjusted for crime or degree of urbanisation. Although populations can be segregated by race, religion or socioeconomic status, most US segregation studies have focused on urban racial segregation.^3^ ^9^ ^10^ UK studies have focused on ethnic density and not segregation, with only one study in Northern Ireland suggesting that increased residential segregation leads to increased rates of poor mental health.^11^ However, this study relied on area-based levels of costed utilisation of antidepressant and anxiolytic medications as the indicator of population mental health and did not adjust for individual-level characteristics risking ecological fallacy. In addition, as medication costs vary greatly by drug brand, costs may not be directly attributable to magnitude of prescribing causing noise in the data.

**Figure 1:**
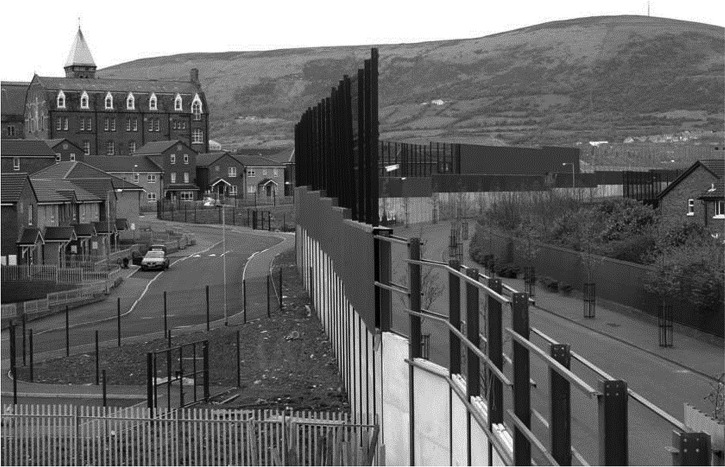
Segregation barrier in West Belfast.

Northern Ireland is a country segregated along religious lines, with religion as the primary geographical divide.^12^ Unlike racial segregation in the USA which stemmed from segregation of new immigrants, segregation in Northern Ireland was amplified in the 1970s, during the civil conflict known colloquially as ‘the Troubles’. Northern Ireland has been largely segregated into Protestant and Catholic communities since the 1900s, but after fighting erupted between opposing Nationalist (majority Roman Catholic) and Unionist (majority Protestant faith) populations in the late 1960s, segregation of these two communities intensified, resulting in what was the largest forced population movement in Western Europe since the aftermath of World War II at the time, it was supported by the local government and enshrined in social housing policies.^13^ ^14^ This residential segregation is reinforced in the wider community as almost all children (93%) are educated in segregated schools promoting very little contact with the ‘other’ community.^15^ Physical manifestations of segregation still remain in the form of dividing walls or segregation barriers, known as ‘peacelines’, constructed at interfaces to keep opposing groups or communities separate (figure 1). The majority of segregation barriers are clustered in highly urban areas with around 100 documented permanent and temporary barriers in the capital city of Belfast alone.^16^

Psychologists have reflected on the negative psychological implications of segregation barriers such as the wall separating the West Bank from Israel in the Middle East and the ‘wall effect’ or reactive depression caused by the Berlin Wall;^17^ ^18^ however, no empirical evidence exists regarding the impact these sociopolitical environments have on the health and well-being of the population. Segregated areas lack social cohesion and residential stability with a steeper population decline compared with the rest of Northern Ireland,^19^ and have increased levels of violence, which are all deleterious to mental health.^20^ Segregation barriers are erected with an aim to reduce conflict and violence and create a sense of protection.

The most commonly used formal measure of residential segregation is the Dissimilarity Index, which essentially identifies the proportion of a group that would need to move in order to create a uniform distribution of the population.^11^ However, increasing literature on segregation has led researchers to debate the accuracy and effectiveness of this measure.^21^ ^22^ The modifiable areal unit problem (MAUP) arises from a reliance on residential population data that are typically collected, aggregated and reported for spatial units that may not accurately conceptualise neighbourhoods. The checkerboard problem stems from the fact that segregation measures typically ignore the spatial proximity of neighbourhoods and focus instead only on racial composition. Northern Ireland is unique in that it contains tangible, physical manifestations of segregation in the form of segregation barriers which can be used as proxies to accurately identify segregated areas, overcoming the MAUP and allowing for a more accurate identification of interface areas with a close proximity to the ‘other’ community. This study will use both the formal Dissimilarity Index and proximity to a segregation barrier to test the association between segregation and poor mental health utilising population-wide linkage of individual records from administrative data sources.

The study aims to determine if segregation is associated with mental health, if the association differs depending on the use of the formal or proxy measure of segregation and if these associations can shed any light on the mechanisms underlying the association between place of residence and mental health. This is the first study of its kind to examine the effect of residential religious segregation on individual mental health in a full population cohort.

## Methods

### Data sources

This was a population-based, record linkage study involving data from the National Health Applications and Infrastructure Services (NHAIS) data set linked to prescribing data on antidepressant and anxiolytic medications from the Enhanced Prescribing Database (EPD) and area-level measures of segregation, deprivation and crime. NHAIS contains information on all patients registered with a primary care physician in Northern Ireland including individual health and care number (HCN—a unique identifier for use within the health system), demographic details, address information and details of the prescribing general practice (GP). Northern Ireland has a universal, free at the point of service healthcare system providing healthcare to almost 100% of the population, and hence almost the entire population are registered with a primary care physician. The EPD is a centralised collation of all medications dispensed to the Northern Ireland population in community pharmacies from 2008 onwards,^23^ and also contains individual HCN facilitating a one-to-one linkage to the NHAIS data set.

### Cohort description

The study cohort consisted of all non-institutionalised individuals living in Northern Ireland aged between 18 and 74 years in 2009. The age restriction allowed for a more accurate identification of poor mental health as antidepressant and anxiolytic medications are sometimes used for indications other than mental illness in the very young and very old.^24–26^ Age was categorised as 18–24, 25–34, 35–44, 45–54, 55–64 and 65–74 years. Address was available for all cohort members and was used to identify area-level characteristics.

### Area characteristics

Census output areas (COAs) were introduced in Northern Ireland after the 2001 Census and were built from clusters of adjacent postcodes (aka zip codes). They have population sizes of around 125 households/350 people. There are 5022 COAs in Northern Ireland. Groups of five or six COAs make up the larger, most commonly used area identifier in the reporting of national geography, the super output area (SOA). In total, Northern Ireland is made up of 890 SOAs with an average population of 2000 people. SOAs are the optimal small area geography for reporting results as they have been designed to be as similar as possible in population size while being big enough to ensure robust estimates of area-level characteristics. Address information from NHAIS was used to assign individuals to COAs and subsequent SOAs.

### Segregation

The Dissimilarity Index, D, of segregation was constructed for each SOA by calculating the proportion of individuals in each sub area (COA) that would have to move in order that the two communities, Catholic and Protestant, were spread evenly throughout the area, also taking into consideration population distribution of SOAs contiguous. More detail of the measure can be found in French (2009).^11^ A dichotomous variable identified areas as segregated using a benchmark of a D ≥0.6. Generally, a Dissimilarity Index value above 0.60 is thought to represent extremely high segregation.^27^

### Segregation barriers

**Figure 2:**
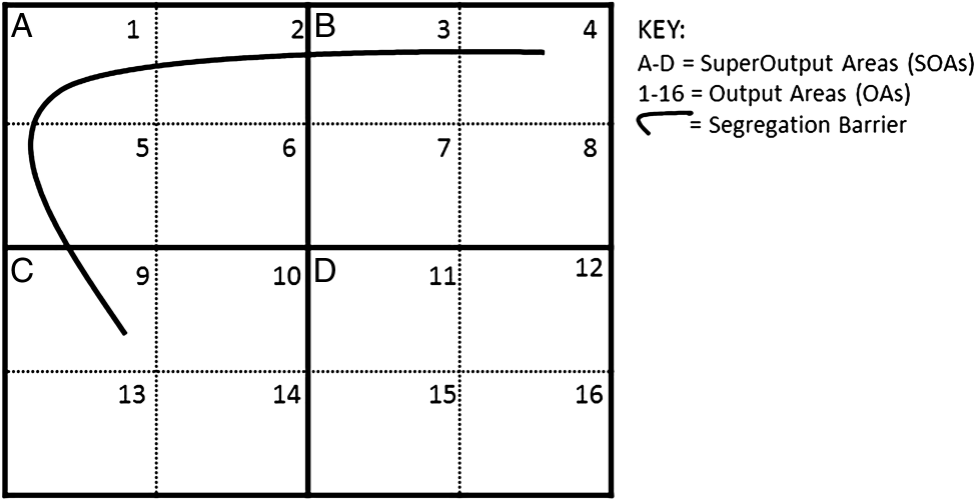
Tabular representation of identifying areas with a segregation barrier.

The Department of Justice (formerly Northern Ireland Office) managed ‘peacelines’ were mapped using Geographical Information Systems technology in 2005 and updated in 2007 by the Belfast Interface Project (BIP) identifying 40 unique segregation barriers in Northern Ireland.^16^ Continuous, unbroken lines of barrier were counted as one ‘peaceline’, so that one barrier made up of cement wall, fencing and wire was counted as one instead of three separate structures. Each of the COAs and SOAs inhabited by a segregation barrier were identified and flagged and used to create an ordinal variable to identify proximity of residence to a segregation barrier (figure 2). Zero indicates no segregation barrier in your area (ie, those living in SOA D in figure 2), one indicates living in close proximity to a segregation barrier (those living in SOA B, COAs 7 and 8 in figure 2), and two indicates living in very close proximity to a segregation barrier (those living in SOA A, COAs 1, 2 and 5 in figure 2).

### Urban-rural

There is no universally agreed definition of what constitutes an ‘urban’ or ‘rural’ area, but an approach based on population size, density and access to services has been used in the UK to produce an official classification of urbanity and this has been applied to the 5022 COAs.^28^ For the purposes of this study, urban was classified as settlements of >75 000 people and rural was classified as settlements of <75 000 people. The urban group encompasses Northern Ireland's two largest cities which are home to almost half the population.

### Deprivation

A measure of disadvantage was extracted from the income deprivation domain of the Northern Ireland Multiple Deprivation Measure (NIMDM), which provides information on the proportion of the population in each area living in households in receipt of income-related benefits and tax credits in 2008/2009.^29^ Scores were ranked and split into quintiles containing approximately equal proportions of the population identifying affluent through to deprived areas. Quintiles were used to plainly illustrate the distribution of segregation and segregation barriers throughout Northern Ireland.

### Crime

Statistics on reported crime in 2008/2009 were also sourced from the NIMDM. The crime domain includes counts of recorded crime, deliberate fires and incidents of antisocial behaviour from the Police Service for Northern Ireland and the Northern Ireland Fire and Rescue Service. Scores were ranked and split into quintiles containing approximately equal proportions of the population identifying areas with high levels of reported crime through to areas with lower levels of reported crime. Though the reporting of crime may be subject to community bias, this is the only measure of crime available.

### Mental health outcomes

Previous studies in this area have relied on self-reported measures of mental health. These are subject to a variety of biases, with self-rated mental health suggested to be more of a measure of well-being than of subjective mental illness,^30^ and with validated scales still being subject to responder and interviewer bias. In this study, receipt of anxiolytic or antidepressant medication was taken as a proxy measure of anxiety disorder or depression, identifying individuals who have sought help for a common mood disorder that is likely affecting their everyday life. Prescription information was retrieved from the EPD for all dispensed anxiolytic and antidepressant medications (British National Formulary (BNF) category 4.1.2 and 4.3) which are predominantly prescribed for depression and anxiety disorders,^31^ ^32^ over the 24-month study period, October 2008 to September 2010. This exceptional data set allows for a detailed examination of prescribing at a nationwide level. Two years’ of data allowed for the identification of long-term users ruling out one-off prescriptions for transient events. Individuals were identified as anxiolytic or antidepressant medication users if they received at least three prescriptions for each drug over the study period. Sensitivity analyses were carried out using (1) a cut-off of at least six prescriptions and (2) categorising ever versus never use, yielding similar results (available on request).

### Data linkage

The prescribing data were linked to the NHAIS using the unique HCN. Linkages were undertaken by the data custodians at the Business Services Organisation and the resultant research data set containing only fully anonymised data was made available to the research team. The study was approved by the Office for Research Ethics Committees Northern Ireland (ORECNI no: 10/NIR02/21).

### Analytic approach

Analysis was divided into three stages. The first, descriptive analysis of the cohort to determine the demographic profile of the residents and the prevalence of medication use in individuals who were resident in segregated areas, either defined by the Dissimilarity Index or by proximity to segregation barriers, compared with that of the rest of the population. Crosstabs of dissimilarity and segregation barriers were completed to determine the potential for mismeasurement. The second stage of analysis involved the construction of two-level multilevel logistic regression models to quantify the association between segregation measured by the Dissimilarity Index and prescription drug uptake, adjusting for intrapractice variation. As the data deal with prescription information, individuals will be naturally clustered within the 356 GPs that service these areas and trends may differ between practices. Some evidence exists to suggest particular practice characteristics can affect the likelihood of prescribing.^33^ Models were built to adjust for age, gender, urbanicity, deprivation and levels of crime and disorder. Interactions between independent variables were tested for moderation effects based on strong suggestions from the descriptive analysis. Third, multilevel logistic regression models were constructed to determine the effect of residential proximity to a segregation barrier and likelihood of anxiolytic or antidepressant usage, adjusting for intrapractice variation. All analyses were carried out in STATA V.13.0.

## Results

The study cohort consisted of 1 323 363 individuals aged between 18 and 74 years in 2009 nested within 356 GPs. The majority of the Northern Irish population lives in non-segregated areas with 23.0% of the population living in areas defined as segregated with a Dissimilarity Index score ≥0.6, and 4.5% of the population living in areas with a segregation barrier or ‘peaceline’ (table 1). For the purposes of presentation in this table, proximity to a segregation barrier is dichotomised. Of those areas identified as having a segregation barrier, 93.6% also scored >0.6 on the Dissimilarity Index, indicating the D is accurately capturing segregated areas.

**Table 1:** Percentage of the Northern Irish population aged 18–74 years (1 323 363) by demographic and area-level characteristics per segregation category

		Segregated areas (as defined by D >0.6)		Segregated areas (as defined by barriers)	
	Total population N=1 323 363	Segregated n=316 485	Non-segregated n=1 058 393	Barrier n=61 942	No barrier n=1 312 936
Gender					
Male	50.2	50.7	50.1	51.3	50.2
Female	49.8	49.3	49.9	48.8	49.8
Age (years)					
18–24	14.8	15.6	14.5	17.1	14.6
25–34	20.8	22.3	20.3	23.2	20.6
35–44	21.0	20.9	21.1	19.6	21.1
45–54	18.9	18.3	19.0	18.1	18.9
55–64	14.4	13.3	14.7	12.4	14.5
65–74	10.3	9.6	10.5	9.6	10.3
Conurbation					
Urban	38.9	52.1	34.9	91.5	36.4
Rural	61.1	47.9	65.1	8.5	63.6
Deprivation					
1 (least)	20.9	7.7	24.8	0.0	21.8
2	22.0	16.4	23.7	0.0	23.0
3	17.7	14.9	18.6	0.0	18.6
4	20.1	21.8	19.6	12.9	20.5
5 (most)	19.3	39.2	13.4	87.1	16.1
Crime					
1 (least)	19.9	16.6	20.9	0.0	20.8
2	20.1	13.6	22.1	0.0	21.1
3	20.0	12.9	22.1	8.3	20.5
4	19.8	25.3	18.2	22.1	19.7
5 (most)	20.2	31.6	16.7	69.6	17.8
Segregation index					
Dissimilarity Index >0.6	23.0	–	–	93.6	19.7
Dissimilarity Index <0.6	77.0	–	–	6.4	80.3
Segregation barrier					
No barrier	95.5	19.7	80.3	–	–
Close or very close to barrier	4.5	93.6	6.4	–	–
Medication					
Antidepressants	13.9	15.6	13.5	20.5	13.6
Anxiolytics	4.3	5.2	4.0	8.6	4.1
Either drug	15.5	17.3	14.9	23.3	15.1

There was no significant difference in the age and gender distribution of those living in segregated or non-segregated areas. Segregated areas tended to be more urban, more deprived and have higher levels of crime compared with those non-segregated areas. Segregation barriers were unique to deprived areas with all barriers in deprivation quintiles 4 and 5, whereas segregation defined by the Dissimilarity Index was evident in all areas, but with more than half (61%) of areas scoring D ≥0.6 falling into deprivation quintiles 4 and 5.

Individuals living in segregated areas, either defined by the Dissimilarity Index or proximity to segregation barriers, had poorer mental health compared with those who did not live in segregated areas. In segregated areas defined by the Dissimilarity Index, 15.6% of the population received ≥3 prescriptions for antidepressant medication and 5.2% received ≥3 prescriptions for anxiolytic medication compared with 13.5% and 4.0%, respectively, in non-segregated areas. Over one in five (20.5%) of those living in areas with segregation barriers received ≥3 prescriptions for antidepressant medication compared with 13.6% of those in areas with no barriers, and 8.6% received ≥3 anxiolytic prescriptions compared with 4.1% in areas with no barriers. The percentage uptake of medication in non-segregated areas defined either way was equivalent.

### Segregation as measured by D and mental health

Separate multilevel logistic models were constructed to determine the likelihood of antidepressant medication, anxiolytic medication or either medication after adjusting for individual factors, urbanicity, deprivation and level of crime. Although the trends were similar, they varied in magnitude for antidepressant and anxiolytic medication, so the results are presented for each medication separately. Table 2 illustrates the likelihood of antidepressant medication given residence in a segregated area as defined by the Dissimilarity Index. In the unadjusted model, individuals living in segregated areas are 7% (OR=1.07, 95% CI 1.06 to 1.09) more likely to receive medication for depression compared with those in non-segregated areas. Adjusting for age, sex and level of urbanity does not alter the association. However, after adjusting for area-level deprivation (model 4) the association disappears (OR=1.00, 95% CI 0.98 to 1.01). The same pattern is observed in analysing the likelihood of anxiolytic medication (table 3). In the model adjusted for age, sex and conurbation (model 3), individuals living in segregated areas are 18% (OR=1.18, 95% CI 1.15 to 1.20) more likely to receive medication for anxiety compared with those in non-segregated areas, but after adjusting for deprivation the association fails. Sensitivity analysis building models using the Dissimilarity Index scores in place of the dichotomised variable, allowing Dissimilarity to vary continuously, yielded similar results. In the unadjusted model, likelihood of antidepressant medication increased significantly with every unit increase in Dissimilarity (β=0.022, p<0.001); however, after adjusting for area-level deprivation, the coefficient loses significance (β=−0.0037, p=0.107). Full results available on request.

**Table 2:** Multilevel logistic regression calculating likelihood of ≥3 antidepressant medication prescriptions given area-level dissimilarity, adjusting for GP variation

	Model 1	Model 2	Model 3	Model 4	Model 5
Segregation					
D <0.6	1.00	1.00	1.00	1.00	1.00
D >0.6	1.07 (1.06 to 1.09)	1.10 (1.08 to 1.11)	1.09 (1.07 to 1.11)	1.00 (0.98 to 1.01)	1.00 (0.98 to 1.01)
Gender					
Male		1.00	1.00	1.00	1.00
Female		2.28 (2.25 to 2.30)	2.28 (2.25 to 2.30)	2.29 (2.27 to 2.32)	2.29 (2.27 to 2.32)
Age (years)					
18–24		1.00	1.00	1.00	1.00
25–34		1.92 (1.87 to 1.97)	1.92 (1.87 to 1.97)	1.91 (1.87 to 1.96)	1.91 (1.87 to 1.96)
35–44		3.39 (3.32 to 3.47)	3.40 (3.32 to 3.48)	3.48 (3.40 to 3.58)	3.48 (3.40 to 3.56)
45–54		4.36 (4.26 to 4.46)	4.36 (4.26 to 4.46)	4.49 (4.39 to 4.60)	4.49 (4.39 to 4.60)
55–64		4.69 (4.58 to 4.80)	4.70 (4.59 to 4.81)	4.83 (4.72 to 4.94)	4.83 (4.72 to 4.95)
65–74		3.76 (3.67 to 3.85)	3.76 (3.67 to 3.86)	3.83 (3.74 to 3.93)	3.83 (3.74 to 3.93)
Conurbation					
Rural			1.00	1.00	1.00
Urban			1.24 (1.20 to 1.27)	1.08 (1.05 to 1.11)	1.07 (1.04 to 1.10)
Deprivation					
1—most affluent				1.00	1.00
2				1.20 (1.18 to 1.22)	1.19 (1.17 to 1.21)
3				1.40 (1.37 to 1.42)	1.38 (1.35 to 1.40)
4				1.66 (1.63 to 1.69)	1.62 (1.59 to 1.66)
5—most deprived				2.06 (2.02 to 2.10)	2.02 (1.97 to 2.07)
Crime					
1—low crime					1.00
2					1.04 (1.02 to 1.06)
3					1.05 (1.03 to 1.07)
4					1.06 (1.04 to 1.08)
5—high crime					1.05 (1.03 to 1.08)

Figures represent ORs and 95% CIs.

Model 1: unadjusted.

Model 2: adjusted for gender and age.

Model 3: plus adjustment for urban/rural.

Model 4: plus adjustment for area level deprivation.

Model 5: plus adjustment for reported crime.

GP, general practice.

**Table 3:** Multilevel logistic regression calculating likelihood of ≥3 anxiolytic medication prescriptions given area-level dissimilarity, adjusting for GP variation

	Model 1	Model 2	Model 3	Model 4	Model 5
Segregation					
D <0.6	1.00	1.00	1.00	1.00	1.00
D >0.6	1.16 (1.13 to 1.19)	1.19 (1.16 to 1.22)	1.18 (1.15 to 1.20)	1.02 (1.00 to 1.05)	1.03 (1.00 to 1.05)
Gender					
Male		1.00	1.00	1.00	1.00
Female		1.91 (1.87 to 1.94)	1.90 (1.87 to 1.94)	1.91 (1.88 to 1.95)	1.91 (1.88 to 1.95)
Age (years)					
18–24		1.00	1.00	1.00	1.00
25–34		2.29 (2.18 to 2.41)	2.29 (2.18 to 2.41)	2.28 (2.17 to 2.39)	2.28 (2.16 to 2.39)
35–44		4.11 (3.92 to 4.31)	4.12 (3.93 to 4.32)	4.26 (4.06 to 4.46)	4.26 (4.06 to 4.47)
45–54		5.50 (5.25 to 5.76)	5.51 (5.26 to 5.78)	5.74 (5.48 to 6.02)	5.75 (5.49 to 6.02)
55–64		6.70 (6.39 to 7.02)	6.72 (6.41 to 7.04)	7.00 (6.68 to 7.34)	7.00 (6.68 to 7.34)
65–74		7.72 (7.36 to 8.10)	7.73 (7.37 to 8.11)	7.96 (7.59 to 8.35)	7.96 (7.58 to 8.35)
Conurbation					
Rural			1.00	1.00	1.00
Urban			1.48 (1.41 to 1.55)	1.20 (1.14 to 1.25)	1.18 (1.12 to 1.24)
Deprivation					
1—most affluent				1.00	1.00
2				1.32 (1.28 to 1.36)	1.27 (1.23 to 1.32)
3				1.62 (1.60 to 1.68)	1.55 (1.49 to 1.60)
4				2.10 (2.03 to 2.17)	1.94 (1.87 to 2.02)
5—most deprived				2.91 (2.82 to 3.01)	2.67 (2.57 to 2.78)
Crime					
1—low crime					1.00
2					1.12 (1.08 to 1.16)
3					1.16 (1.12 to 1.20)
4					1.17 (1.13 to 1.22)
5—high crime					1.22 (1.17 to 1.26)

Figures represent ORs and 95% CIs.

Model 1: unadjusted.

Model 2: adjusted for gender and age.

Model 3: plus adjustment for urban/rural.

Model 4: plus adjustment for area level deprivation.

Model 5: plus adjustment for reported crime.

GP, general practice.

### Segregation as measured by proximity to ‘peacelines’ and mental health

Table 4 illustrates the likelihood of antidepressant medication given residence in a segregated area as defined by proximity to a segregation barrier or ‘peaceline’. As ‘peacelines’ are unique to deprived areas, the more affluent areas were excluded from this analysis to provide stricter, but fairer, comparisons with the unexposed group. A total of 521 970 individuals in Northern Ireland live in the most deprived quintiles (deprivation quintiles 4 and 5), with 7.8% (40 759) of the deprived population living close to a ‘peaceline’ and 3.6% (18 719) living very close to a ‘peaceline.’ The reference category was defined as individuals living in deprived areas with no segregation barrier comparing individuals who lived close to a barrier (barrier at an SOA level) or very close to a barrier (barrier at a COA level). Living close to or very close to a segregation barrier is associated with a 15% increased likelihood of antidepressant medication compared with those living in deprived non-segregated areas in the unadjusted model (OR=1.15, 95% CI 1.11 to 1.20). There is no evidence of a step-wise relationship based on proximity to segregation barrier. Adjustment for all confounders has no effect on the association. In the final model adjusted for sex, age, conurbation and crime, individuals living very close to a segregation barrier are 19% more likely to receive antidepressant medication compared with those living in deprived non-segregated areas (OR=1.19, 95% CI 1.14 to 1.23).

**Table 4:** Multilevel logistic regression calculating likelihood of ≥3 antidepressant medication prescriptions given proximity to segregation barriers, adjusting for GP variation

	Model 1	Model 2	Model 3	Model 4	Model 5
Presence of segregation barrier					
Deprived no barrier (462 492)	1.00	1.00	1.00	1.00	1.00
Deprived barrier SOA (40 759)	1.15 (1.12 to 1.18)	1.18 (1.15 to 1.22)	1.18 (1.14 to 1.21)	1.17 (1.13 to 1.20)	1.17 (1.13 to 1.20)
Deprived barrier COA (18 719)	1.15 (1.11 to 1.20)	1.21 (1.16 to 1.26)	1.20 (1.15 to 1.25)	1.19 (1.14 to 1.23)	1.19 (1.14 to 1.23)
Gender					
Male		1.00	1.00	1.00	1.00
Female		2.29 (2.27 to 2.31)	2.29 (2.27 to 2.31)	2.29 (2.27 to 2.31)	2.29 (2.27 to 2.31)
Age (years)					
18–24		1.00	1.00	1.00	1.00
25–34		1.92 (1.87 to 1.96)	1.92 (1.87 to 1.96)	1.91 (1.86 to 1.96)	1.91 (1.86 to 1.96)
35–44		3.46 (3.38 to 3.54)	3.46 (3.38 to 3.54)	3.47 (3.39 to 3.55)	3.47 (3.39 to 3.55)
45–54		4.45 (4.35 to 4.56)	4.46 (4.36 to 4.56)	4.47 (4.37 to 4.57)	4.47 (4.37 to 4.57)
55–64		4.79 (4.68 to 4.91)	4.80 (4.69 to 4.91)	4.80 (4.69 to 4.92)	4.80 (4.69 to 4.92)
65–74		3.81 (3.72 to 3.91)	3.82 (3.72 to 3.91)	3.82 (3.72 to 3.91)	3.82 (3.72 to 3.91)
Conurbation					
Rural			1.00	1.00	1.00
Urban			1.13 (1.10 to 1.16)	1.10 (1.07 to 1.13)	1.10 (1.07 to 1.13)
Crime					
1—low crime				1.00	1.00
2				1.06 (1.04 to 1.08)	1.06 (1.04 to 1.08)
3				1.10 (1.08 to 1.12)	1.10 (1.08 to 1.12)
4				1.15 (1.13 to 1.21)	1.15 (1.13 to 1.21)
5—high crime				1.19 (1.16 to 1.21)	1.19 (1.16 to 1.21)
Dissimilarity Index					
<0.6					1.00
>0.6					1.00 (0.98 to 1.01)

Figures represent ORs and 95% CIs.

Model 1: unadjusted.

Model 2: adjusted for gender and age.

Model 3: plus adjustment for urban/rural.

Model 4: plus adjustment for reported crime.

Model 5: plus adjustment for Dissimilarity.

COA, census output area; GP, general practice; SOA, super output area.

The pattern for anxiolytic use is much more pronounced (table 5). In the unadjusted model (model 1) individuals living close to a segregation barrier are 25% more likely to receive anxiolytic medication compared with those living in deprived, non-segregated areas (OR=1.25, 95% CI 1.20 to 1.30), and those living very close are 37% more likely to receive anxiolytic medication (OR=1.37, 95% CI 1.30 to 1.45). After full adjustment for sex, age, conurbation and reported crime the association remains. Even after adjustment for level of segregation measured using the Dissimilarity Index, the association between proximity to ‘peaceline’ and poor mental health remains (model 5). Individuals living in deprived segregated areas closest to a segregation barrier are 39% more likely to receive anxiolytic medication than those living in deprived non-segregated areas with no segregation barrier. There is a clear step-wise relationship between proximity to the segregation barrier and likelihood of anxiety medication.

**Table 5:** Multilevel logistic regression calculating likelihood of ≥3 anxiolytic medication prescriptions given proximity to segregation barriers, adjusting for GP variation

	Model 1	Model 2	Model 3	Model 4	Model 5
Presence of segregation barrier					
Deprived no barrier (462 492)	1.00	1.00	1.00	1.00	1.00
Deprived barrier SOA (40 759)	1.25 (1.20 to 1.30)	1.29 (1.23 to 1.34)	1.27 (1.22 to 1.33)	1.25 (1.19 to 1.30)	1.24 (1.18 to 1.29)
Deprived barrier COA (18 719)	1.37 (1.30 to 1.45)	1.45 (1.37 to 1.53)	1.43 (1.36 to 1.52)	1.40 (1.33 to 1.48)	1.39 (1.32 to 1.48)
Gender					
Male		1.00	1.00	1.00	1.00
Female		1.91 (1.88 to 1.95)	1.91 (1.88 to 1.95)	1.91 (1.88 to 1.95)	1.91 (1.88 to 1.95)
Age (years)					
18–24		1.00	1.00	1.00	1.00
25–34		2.28 (2.17 to 2.40)	2.29 (2.17 to 2.40)	2.28 (2.17 to 2.39)	2.28 (2.17 to 2.39)
35–44		4.22 (4.03 to 4.43)	4.23 (4.04 to 4.44)	4.24 (4.05 to 4.45)	4.24 (4.05 to 4.45)
45–54		5.68 (5.42 to 5.95)	5.68 (5.42 to 5.95)	5.71 (5.45 to 5.98)	5.71 (5.45 to 5.98)
55–64		6.92 (6.60 to 7.25)	6.93 (6.61 to 7.26)	6.95 (6.63 to 7.28)	6.95 (6.63 to 7.28)
65–74		7.90 (7.53 to 8.29)	7.90 (7.53 to 8.29)	7.91 (7.54 to 8.30)	7.91 (7.54 to 8.30)
Conurbation					
Rural			1.00	1.00	1.00
Urban			1.28 (1.22 to 1.34)	1.22 (1.17 to 1.28)	1.22 (1.17 to 1.28)
Crime					
1—low crime				1.00	1.00
2				1.14 (1.11 to 1.18)	1.15 (1.11 to 1.19)
3				1.22 (1.18 to 1.26)	1.22 (1.18 to 1.27)
4				1.31 (1.27 to 1.36)	1.31 (1.27 to 1.36)
5—high crime				1.43 (1.38 to 1.48)	1.43 (1.38 to 1.48)
Dissimilarity Index					
<0.6					1.00
>0.6					1.02 (0.99 to 1.04)

Figures represent ORs and 95% CIs.

Model 1: unadjusted.

Model 2: adjusted for gender and age.

Model 3: plus adjustment for urban/rural.

Model 4: plus adjustment for reported crime.

Model 5: plus adjustment for Dissimilarity.

COA, census output area; GP, general practice; SOA, super output area.

## Discussion

This study aimed to determine the risk of poor mental health based on segregation measured both by the Dissimilarity Index and by proximity to segregation barriers in an attempt to understand the mechanisms underlying any association between segregation and mental health. When using the formal Dissimilarity Index as a measure of population segregation, the results suggest that segregation poses no additional risk to the likelihood of poor mental health after adjusting for area-level deprivation. However, when using proximity to a segregation barrier, that is, ‘peaceline’, as a proxy indicator of segregation, the results suggest that living in an area segregated by a ‘peaceline’ increases the likelihood of antidepressant medication by 19% and of anxiolytic medication by 39%, even after adjusting for gender, age, level of crime and conurbation. Segregation barriers are unique to deprived areas. So living in a deprived area with a segregation barrier provides an additional risk for poor mental health over and above the known excess risk of poor mental health in deprived areas. Choice of segregation indicator changed the magnitude of the measured association between segregation and mental health.

### Comparison to other studies

These findings challenge a previous study in Northern Ireland which found that segregation as measured by the Dissimilarity Index had a negative effect on mental ill health,^11^ though, that study relied on coarse area-level measures of antidepressant and anxiolytic prescription costs and, unlike the present work, did not adjust for individual characteristics, individual-level uptake of medications or GP variation. In the current study, adjusting for deprivation attenuated the association between area-level segregation, as measured by D, and mental health, supporting the premise that deprivation is the major determinant of mental health.^34^

The findings from this study raise a number of concerns surrounding the conceptualisation of segregation. Individuals living in areas with a segregation barrier are without doubt segregated, but it may be the built environment and the segregation infrastructure, not population composition per se, that is affecting mental health in these areas.

### Dissimilarity Index versus ‘peacelines’

Segregation as measured by the Dissimilarity Index measures the distribution of the population in an area but tells us little about how the population use this space or how likely individuals are to interact with the ‘other’ group. Segregation as measured by proximity to a ‘peaceline’ not only illustrates how a population is distributed over space, but also highlights the physical presence of a barrier in the built environment and the physical division of communities preventing interaction with the ‘other’ group, which some commentators have proposed is more indicative of segregation than the distribution of groups in communities.^35^ ^36^ Four mechanisms have been suggested as plausible mediating pathways for the observed association between segregation and poor health outcomes: (1) residential segregation begets individual socioeconomic status; (2) segregation perpetuates unhealthy environments; (4) segregation modifies social capital; and (4) segregation modifies risk behaviours or exposure to stressful stimuli.^21^ This study illustrates that the effect of segregation as measured by the Dissimilarity Index on mental health is mediated by neighbourhood socioeconomic status. However, the effect of segregation as measured by proximity to ‘peacelines’ on mental health is attenuated but not fully explained by neighbourhood socioeconomic status, neighbourhood crime or segregation as measured by the Dissimilarity Index. Based on the four aforementioned mechanisms this would suggest proximity to a ‘peaceline’ may modify risk behaviours or exposure to stressful stimuli.

The likelihood of receiving medication for anxiety disorders was higher in areas defined by segregation barriers than the likelihood of receiving medication for depression, suggesting the stress or anxiety provoking nature of the environment. The protective walls and barriers between communities in Northern Ireland have become a focal point for low-level and localised violence, which may not be captured in the formal crime and disorder measure. A sense of permanent threat in these areas as ongoing sectarian violence exacerbates social, psychological and environmental difficulties may contribute to the likelihood of receiving anxiolytic medication.^37^ Therefore, residential segregation may affect risk of anxiety disorder more so than risk of depression. In addition, historically, these areas witnessed most of the politically motivated violence associated with the ‘troubles’.^38^ Evidence suggests that the likelihood of psychological morbidity increases the greater the extent to which the troubles affected a person's area or life.^39^ However, for events from the past to affect current mental health status, we would have to assume little migration from these areas and in addition, many younger residents were born after the peace process and are unlikely to have been directly affected. Furthermore, individuals identified as suffering conflict-related trauma are not exclusively located in those areas characterised by intense violence during the ‘troubles’.^40^ It is difficult to disentangle the effect of segregation on mental health for those who live close or very close to ‘peacelines’ from the historical impact of the troubles, ongoing sectarian violence and the effect the barriers have on the landscape.

Recent research on racial segregation has also focused on the effect of perceived discrimination. A systematic review of self-reported racism and health found a negative association with mental health even after adjustment for confounders.^41^ The majority of the reviewed studies (86%) were based in the USA but an English study of 2054 employed adults found strong associations between perceived racial/ethnic discrimination and common mental disorders.^42^ Perceived discrimination was not measured in this study and the higher prevalence of poor mental health in segregated areas defined by both the Dissimilarity Index and proximity to segregation barriers may be due in part to the degree of perceived discrimination.

It is important to note that physical manifestations of segregation, such as barriers, also affect the lived environment in terms of street connectivity and access to resources which are evidenced as being associated with poor mental health.^20^ ‘Peaceline’ areas also contain a lot of graffiti and derelict buildings which have been suggested as negative neighbourhood attributes which can have a deleterious effect on mental health.^43^ These factors were not adjusted for in the current study and may go some way in explaining the observed association between poor mental health and residential proximity to ‘peacelines’.

### Potential limitations

This data linkage project captures information on the entire Northern Ireland population aged 18–74 years and thus has all the advantages of a population-wide study. However, it does rely on arbitrary geographic identification of areas which may not accurately conceptualise neighbourhoods, though the area measures with approximately 350 people, contain finer detailed information than previous work which has relied on census tract delineations of up to 9000 people.^4^ In addition, our measure of proximity to a ‘peaceline’, like all segregation measures, is aspatial and it is plausible that someone in a neighbouring SOA actually lives closer to the barrier than someone in the SOA depending on where the barrier is located. However, this does not alter the observed associations. Given the polarised nature of the Northern Irish society in terms of the spatial distribution according to religious affiliation, there are potentially many more segregated ‘interface’ areas that are not characterised by a separation barrier. For example, communities have also been separated by urban design in terms of road widening and motorway construction, but these forms of barrier differ substantially from the ‘peaceline’ which is the focus of this paper. In addition, ‘peacelines’ themselves may in fact have a protective effect at these interface areas and were the barrier to be removed mental health may decline. This is a cross-sectional study and it is difficult to determine whether the barriers were built as a result of tensions in the area or if the building of the barriers has in fact contributed to tensions.

## Conclusion

This is the first study of its kind to document individual-level anxiolytic and antidepressant medication uptake of those living in highly segregated areas in Northern Ireland. Living in a segregated area defined by proximity to a ‘peaceline’ is associated with a detrimental effect on mental health. Residential segregation defined by the Dissimilarity Index appears to pose no additional risk to mental health after adjustment for deprivation. This supports the theory that residential segregation is likely to play a role in determining socioeconomic status; hence, adjustment for level of deprivation attenuates the observed relationship between segregation and mental ill health. It may be that community tensions and the heightened sense of ‘other’ in ‘peaceline’ areas are more indicative of poor mental health than segregation itself. Residence in segregated areas with high tensions or at interface areas close to the ‘other’ community may be more detrimental to mental health than residence in segregated areas without these attributes.

This study also highlights the importance of choice of segregation index when measuring the effect of segregation on mental health and supports demands for a more rigorous appraisal of commonly used segregation measures.^4^ In future studies, the environmental expression of segregation (walls, barriers, neighbourhood degeneration) needs to be taken into account and adjusted for to determine the mechanism underlying the association between segregation and health. In Northern Ireland, local policy makers are campaigning for the segregation barriers to be removed permanently.^44^ Although the current study cannot guide policy on whether the walls should remain or come down, their removal would provide an excellent opportunity for a natural experiment examining the impact of barrier removal on mental health. In a research report into attitudes towards the ‘peacelines’, although 58% of the population stated they would like to see the walls come down sometime in the future, 69% maintained that the segregation barriers are still necessary because of the potential for violence.^45^ Physical manifestations of segregation, community tensions and a heightened sense of ‘other’ more so than segregation itself may impact population mental health and future studies should systematically assess the associations between the multiple conceptualisations of segregation.

**What is already known on this subject**

- Neighbourhood segregation has been described as a fundamental determinant of physical health, but literature on its effect on mental health is less clear.
- The majority of literature to date focuses on US racial segregation and recently queries have been raised about the accuracy of segregation measures.
- No UK study exists which has looked at religious residential segregation and mental health, and Northern Ireland provides a unique opportunity for segregation research.

**What this study adds**

- Nationwide Northern Irish data on over 1.3 million individuals suggest residence in an area segregated by a dividing wall, or ‘peaceline’, is deleterious to mental health.
- Residential segregation as measured by the Dissimilarity Index poses no additional risk to mental health after adjustment for socioeconomic status.
- Physical manifestations of segregation, community tensions and a heightened sense of ‘other’ more so than segregation itself may impact population mental health and future studies should systematically assess the associations between the multiple conceptualisations of segregation.
